# Impact of an Asymmetric Cellulose Triacetate (ATA) Membrane on Interleukin-6 Clearance and Pressure Stability During Continuous Hemofiltration in an Ex Vivo Sepsis Model

**DOI:** 10.7759/cureus.92965

**Published:** 2025-09-22

**Authors:** Kouhei Ono, Yoshifumi Ohchi, Shigekiyo Matsumoto, Takenori Makino, Takaaki Kitano

**Affiliations:** 1 Department of Clinical Engineering, Oita University Hospital, Yufu City, JPN; 2 Department of Anesthesiology and Intensive Care, Faculty of Medicine, Oita University, Yufu City, JPN; 3 Department of Anesthesiology, Akeno Central Hospital, Oita City, JPN; 4 Department of Anesthesiology, Shin Beppu Hospital, Beppu, JPN

**Keywords:** asymmetric cellulose triacetate, blood purification, continuous hemofiltration, cytokine removal, sepsis

## Abstract

Background: Asymmetric cellulose triacetate (ATA) membranes have been developed to improve filtration performance and biocompatibility in blood purification therapy. However, their efficacy in cytokine removal and their long-term durability have not been comprehensively evaluated.

Methods: An ex vivo sepsis model was established by incubating human whole blood samples with lipopolysaccharide for 12 hours. Two hemofilter types, ATA and conventional cellulose triacetate (CTA), were compared using a continuous hemofiltration circuit. Interleukin (IL)-6 levels and albumin clearance were measured over 24 hours. The intracircuit pressure was monitored to evaluate the membrane durability.

Results: The ATA membrane achieved significantly higher IL-6 clearance (mean 20.7 (IQR, 17.5-24.5) mL/minute) than the CTA membrane (mean 12.6 (IQR, 10.2-14.9) mL/minute; p = 0.0125). The ATA membrane also demonstrated greater albumin clearance than the CTA membrane (mean 0.74 (IQR, 0.42-0.92) mL/minute vs. mean 0.09 (IQR, 0.07-0.10) mL/minute; p = 0.0039). The ATA membrane demonstrated more stable transmembrane pressure profiles than CTA membranes across the 24-hour observation period.

Conclusion: ATA membranes exhibit enhanced cytokine removal capacity and favorable pressure stability compared with conventional CTA membranes. These findings suggest the potential clinical utility of ATA membranes in conditions requiring efficient mediator clearance, although albumin loss should be carefully considered.

## Introduction

Sepsis is a life-threatening syndrome defined by a dysregulated host response to infection, resulting in severe organ dysfunction [1]. Acute kidney injury (AKI) is widely recognized in patients with sepsis or septic shock and has a poor prognosis [2]. Acute blood purification therapy is frequently utilized in the intensive care unit to support renal function in patients with AKI. Furthermore, excessive production of inflammatory mediators, such as cytokines, can cause organ damage in septic shock, and acute blood purification therapy is reportedly valuable in the treatment of septic shock from the viewpoint of mediator control [3].

Hemofilters used in blood purification therapy are composed of semi-permeable hollow fiber membranes, which have various characteristics depending on the membrane material. Cellulose triacetate (CTA), a membrane material, offers advantages such as high biocompatibility, high antithrombogenicity, and resistance to circuit coagulation, although its performance in removing medium-sized molecules remains inferior to that of other membranes [4,5]. Asymmetric CTA (ATA), a newly developed membrane based on CTA, has an asymmetric dual-layer structure comprising a thin, dense layer that defines filtration performance and a support layer that maintains strength. Compared with conventional CTA membrane, the ATA membrane is expected to improve permeability and antithrombotic properties by reducing protein adhesion within the membrane [6].

Although ATA membranes are hypothesized to outperform conventional CTA membranes in cytokine removal, their efficacy and 24-hour durability remain insufficiently characterized. This study compared ATA and CTA membranes in an ex vivo human sepsis model, focusing on IL-6 and albumin clearance and membrane durability. To our knowledge, this represents the first direct comparison of these membranes in this context.

## Materials and methods

This study was conducted at Oita University Hospital, Yufu City, Oita, Japan, from December 1, 2019, to March 31, 2022. The study was approved by the Ethics Committee of the Faculty of Medicine, Oita University (Approval No. 1715). All participants were informed of the purpose and procedures of the study in writing, and written consent was obtained from each participant.

Participants (volunteer donors)

All participants were Japanese volunteers affiliated with Oita University Hospital. There were no sex-based inclusion criteria. Eligible participants were healthy adults (aged 18 years or older) who were not taking any regular medications, had no infectious diseases or fever, and had not used steroids or nonsteroidal anti-inflammatory drugs within one week prior to blood collection.

Blood collection and cytokine induction

A total of 400 mL of whole blood was collected from healthy volunteer donors in a bag (Carmi® CA solution 400 mL; SB-KAWASUMI LABORATORIES, INC., Kawasaki, Kanagawa, Japan). Lipopolysaccharide (from *Escherichia coli* O127:B8; MilliporeSigma, Burlington, Massachusetts, United States) was added at a concentration of 30 mg per 400 mL to induce inflammatory mediator production. The mixture was incubated at 39°C for 12 hours to induce inflammatory mediator release, thereby establishing the ex vivo sepsis..

Hemofilters and hemofiltration circuits

**Figure 1:**
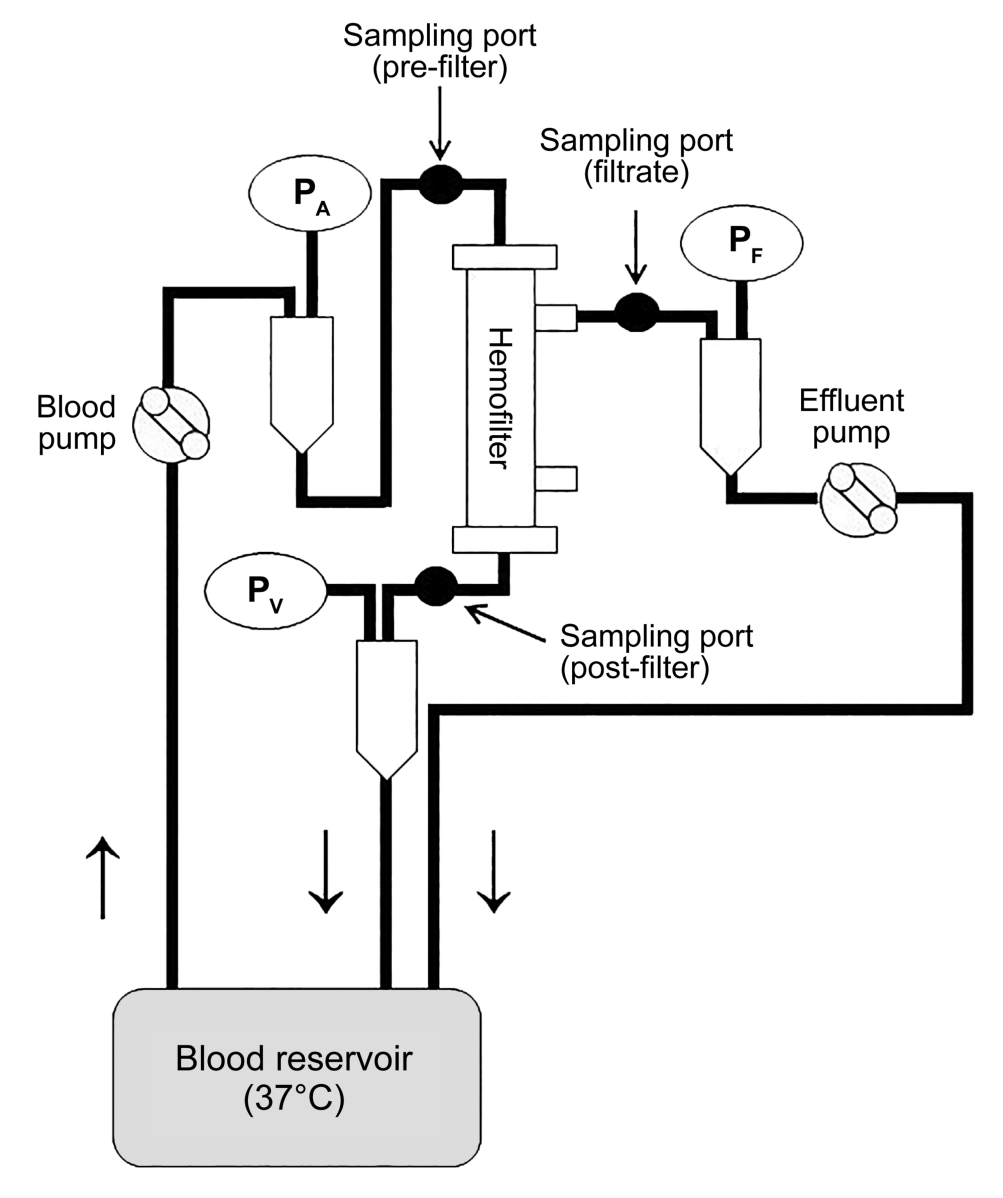
Circuit set-up. P_A_: pre-filter pressure; P_V_: post-filter pressure; P_F_: filtrate pressure

The blood purification circuit was prepared based on previous studies using the same sepsis model [4,5]. A closed blood purification circuit was constructed by setting up a blood purification system (JUN 55X, JUNKEN MEDICAL Co., Ltd., Tokyo, Japan) with a blood circuit and a hemofilter (Figure 1). ATA membrane (AUT-21eco®, Nipro Corporation, Settsu, Osaka, Japan) and conventional CTA membrane (UT-2100S®, Nipro Corporation) were used as hemofilters. The characteristics of the two types of hemofilters are listed in Table 1. The blood flow rate was 150 mL/minute, and the filtration flow rate was 2000 mL/hour for 24 hours. Blood samples were collected from the inlet, outlet, and filtrate ports at six time points: baseline (0 hour) and at one, four, eight, 12, and 24 hours. The samples (inlet and outlet) were centrifuged at 10,000 rpm for 10 minutes at 4°C, and the serum component was divided into two portions of 500 μL each for IL-6 and albumin measurements. The serum was then stored frozen at -80°C. The filtrate sample was divided into microtubes in 1 mL portions and stored frozen at -80°C.

**Table 1:** Differences in characteristics of each membrane type ATA: asymmetric cellulose triacetate; CTA: cellulose triacetate.

	CTA	ATA
Material	Cellulose triacetate	Cellulose triacetate
Inside diameter	200 μm	200 μm
Thickness	15 μm	25 μm
Structure	Homogeneous	Asymmetric
Surface area	2.1 mm^2^	2.1 mm^2^

IL-6 and albumin measurements

IL-6 levels were detected using the Bio-Plex system (Bio-Rad Laboratories, Inc., Hercules, California, United States). Plasma albumin levels were measured using a multichannel biochemical analyzer (BioLis 15i®; Biolis Medical Corp., Tokyo, Japan), and filtrate albumin levels were measured by immunoturbidimetry (LZ U-ALB®; Eiken Chemical Co., Ltd., Tokyo, Japan).

The sieving coefficient (SC) and clearance (CL) were calculated using the following equations as described previously [4,5]:

$SC = \frac{2 C_{uf}}{C_{i} + C_{o}}$

$CL = SC \times Q_{uf}$

where Ci is the supernatant concentration at the filter inlet (pg/mL), Co is the supernatant concentration at the filter outlet (pg/mL), Cuf is the filtrate concentration (pg/mL), and Quf is the ultrafiltration flow rate (mL/minute). CL was calculated at six timepoints: at the beginning (0 hours) and at one, four, eight, 12, and 24 hours. An approximate curve was constructed from the change in 24-hour CL over time, and the average CL over 24 hours was calculated from the area under the curve.

Measuring the pressure in the circuit

The pre-filter pressure (Figure 1, P_A_), post-filter pressure (Figure 1, P_V_), filtrate pressure (Figure 1, P_F_), and transmembrane pressure (TMP) were measured using a blood purification device. The TMP of the device is calculated using the following equation:

$TMP = \frac{(\text{pre-filter pressure} + \text{post-filter pressure})}{2} - \text{filtrate pressure}$

Statistical analysis

Data values are expressed as the median and interquartile range (IQR). The Wilcoxon rank-sum test was used to compare the 24-hour mean CL between the groups. Friedman's test was used to analyze changes in the intracircuit pressure over time, and a post-hoc test was performed using the Wilcoxon rank-sum test with Bonferroni correction. All statistical analyses were performed using EZR v1.61 (Jichi Medical University, Tochigi, Japan). A p-value of <0.05 was considered statistically significant.

## Results

Donor characteristics

A total of eight blood donors were included in the study. The demographic characteristics of the donors are summarized in Table 2. All participants were male and had no comorbidities, medication use, or evidence of infection.

**Table 2:** Donor characteristics (N=8) IQR: interquartile range

Characteristics	Values
Age (year), n (%)	32 (28-36)
Sex (male), n (%)	8 (100%)
BMI (kg/m^2^), median (IQR)	20.3 (19.5-21.9)
Current smoker, n (%)	2 (25%)
Alcohol use (≥1/week), n (%)	2 (25%)
Comorbidities	None
Medication	None
Recent Infection	None

Measurements of IL-6 and albumin

Measured IL-6 and albumin levels at each time point are shown in Table 3.

**Table 3:** Changes over time in IL-6 and albumin levels IL-6: interleukin-6; ATA: asymmetric cellulose triacetate; CTA: cellulose triacetate; h: hours

		0 h, median (IQR)	1 h, median (IQR)	4 h, median (IQR)	8 h, median (IQR)	12 h, median (IQR)	24 h, median (IQR)
IL-6 (pg/mL)	ATA	2546.5	2938.3	4051.1	4941.3	5020.8	5820.6
		(1291.9‒4490.9)	(1331.4‒4344.8)	(2352.6‒5882.6)	(3107.9‒7161.7)	(2732.6‒9621.9)	(3877.1‒11439.8)
	CTA	2546.5	3082.6	3090.6	3504.6	3680.5	3677.7
		(1291.9‒4490.9)	(1457.0‒5177.5)	(1473.4‒5239.3)	(1755.5‒5681.4)	(2467.0‒6451.2)	(2477.0‒6829.0)
Albumin (g/dL)	ATA	0.0053	0.0067	0.0096	0.0239	0.0304	0.0564
		(0.0048‒0.0079)	(0.0060‒0.0084)	(0.0078‒0.0163)	(0.0171‒0.0313)	(0.0239‒0.0171)	(0.0346‒0.0603)
	CTA	0.0053	0.006	0.0059	0.0057	0.004	0.0041
		(0.0048‒0.0079)	(0.0055‒0.0062)	(0.0036‒0.0066)	(0.0044‒0.0071)	(0.0033‒0.0048)	(0.0026‒0.0074)

Mean CL of IL-6 and albumin

**Figure 2:**
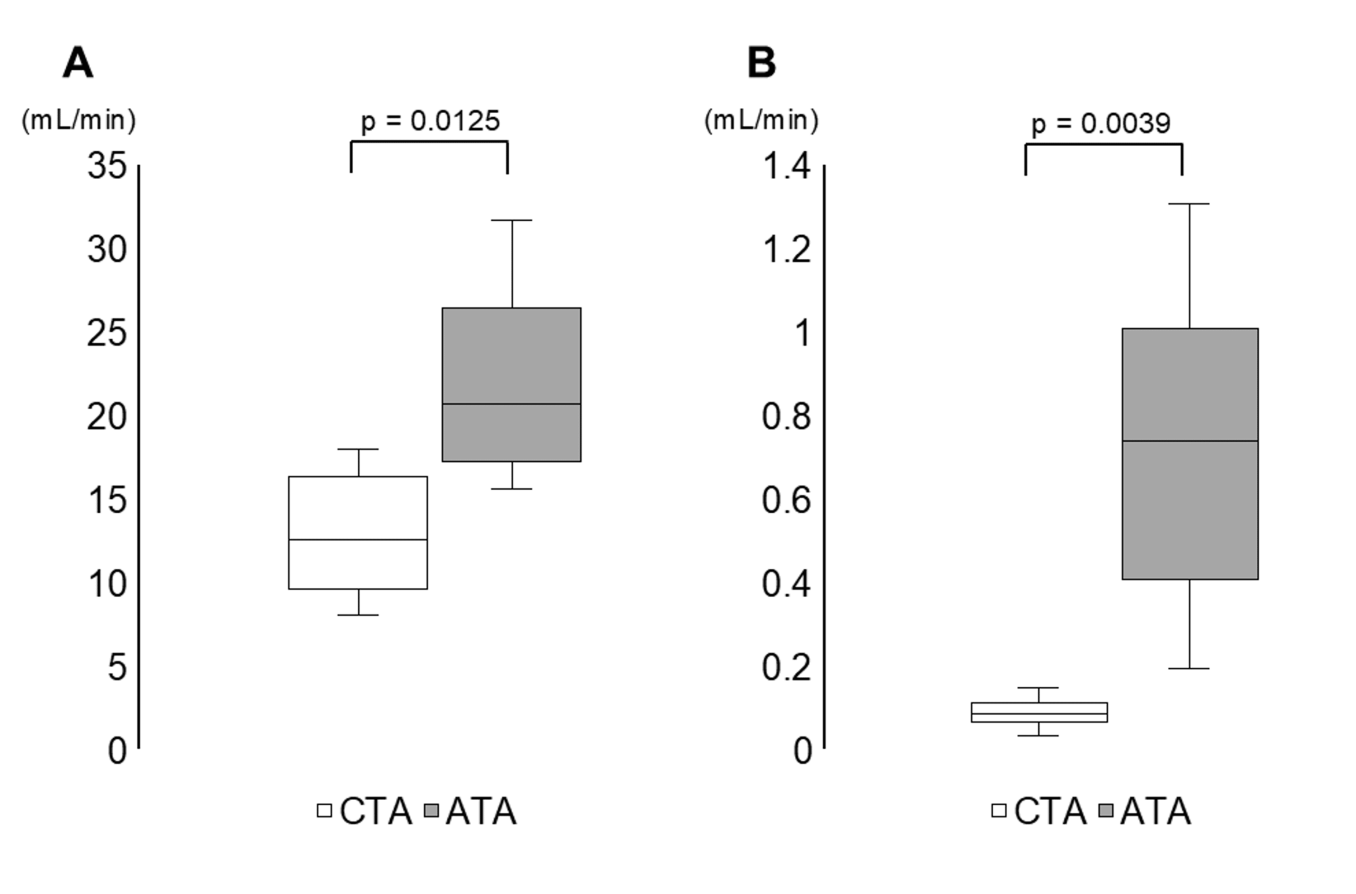
The mean 24-hour clearance of albumin and IL-6. (A) 24-hour mean clearance of IL-6; (B) 24-hour mean clearance of Albumin. IL-6: interleukin-6; ATA: asymmetric cellulose triacetate; CTA: cellulose triacetate

The ATA membrane exhibited a significantly higher mean IL-6 CL (20.7 (IQR, 17.5-24.5) mL/minute) than the CTA membrane (mean 12.6 (IQR, 10.2-14.9) mL/minute; U = 5, p = 0.0125) (Figure 2A). The ATA membrane also demonstrated significantly greater albumin CL (mean 0.74 (IQR, 0.42-0.92) mL/minute) than the CTA membrane (mean 0.09 (IQR, 0.07-0.10) mL/minute; U = 0, p = 0.0039) (Figure 2B).

Time course of intracircuit pressures

**Figure 3:**
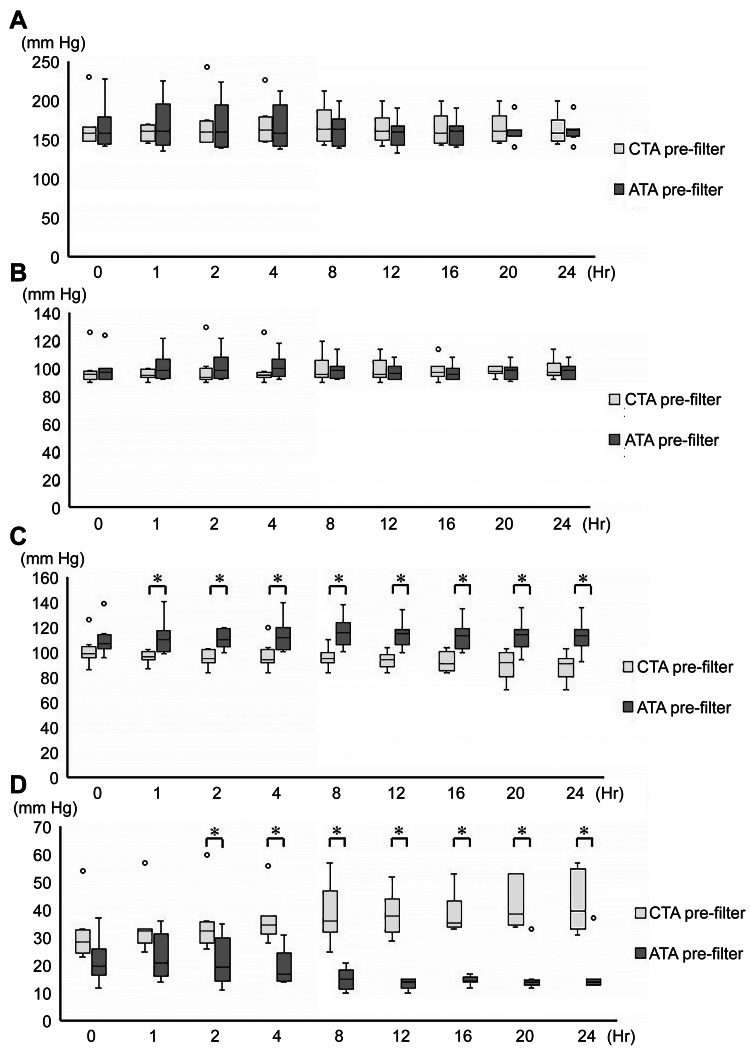
Temporal changes in intracircuit pressures over 24 hours. (A) pre-filter pressure; (B) post-filter pressure; (C) filtrate pressure; (D) transmembrane pressure ＊: p < 0.05 ATA vs. CTA. ATA: asymmetric cellulose triacetate; CTA: cellulose triacetate

No significant temporal variations in intracircuit pressures were observed in either the ATA or CTA groups when compared with baseline values (Figure 3). The pre- and post-filter pressures did not differ significantly between the two groups at any examined time point (Figures 3A, 3B). In contrast, the filtrate pressure was significantly higher in the ATA group than in the CTA group at one hour and remained significantly elevated at all subsequent time points (Figure 3C). TMP was significantly lower in the ATA group beginning at two hours, and the difference persisted at all subsequent time points (Figure 3D).

## Discussion

The current study revealed two main findings. First, ATA membranes demonstrated substantially higher IL-6 and albumin removal capacities than conventional CTA membranes. Second, the ATA membranes exhibited a smaller decrease in filtration pressure, along with a lower increase in TMP, than the CTA membranes.

The removal capacity of the ATA membranes was substantially higher than that of conventional CTA in this study. This difference is attributable to the distinct structure of the ATA membranes compared with conventional CTA, which determines filtration performance. ATA membranes have an asymmetric bilayer structure consisting of a thin, dense layer that defines their filtration performance and a support layer that maintains the strength, whereas conventional CTA membranes have a homogeneous structure [7].

Pores that define the permeability and mass removal capacity reside within the dense layer. The greater the homogeneity of the pores, the higher the mass removal capacity. This was demonstrated experimentally using polyethersulfone membranes with the same asymmetric membrane structure [5]. The ATA membrane has a thin, dense layer with a more homogeneous pore structure and a higher separation capacity for middle-molecular substances than conventional CTA membranes, which restricts the removal of albumin (molecular weight: 65,000 Da) and is thought to remove IL-6 with a relatively large molecular weight (13,000 Da) with high efficiency.

The ATA membrane demonstrated more stable filtration pressures and a lower increase in TMP over time than the conventional CTA membrane in this study. However, there was no significant difference in the pre- and post-filter pressures between the membranes and no change over time. This implies that the resistance of both membranes did not change over time, which may be attributed to reduced thrombus formation and protein adhesion on the surface of the hollow fiber membranes. CTA is a biocompatible material that has been shown to induce minimal platelet stimulation [8] and fibrinogen adhesion [7]. Nonetheless, differences over time were observed only in the filtration pressure and TMP, which may reflect structural differences between the membranes. Conventional CTA membranes have a homogeneous cross-sectional structure, which causes protein accumulation in the membrane compared with those exhibiting an asymmetric structure, with TMP demonstrating a tendency to increase under filtration-based treatment conditions [7]. As described above, the asymmetric dual-layer structure of ATA membranes limits protein accumulation on the membrane surface and prevents protein adhesion to the membrane surface, suggesting that ATA membranes are durable blood purifiers.

The findings suggest that ATA membranes offer superior removal capacity for medium-to-large molecules relative to conventional CTA membranes, though this advantage is offset by greater albumin loss. The high removal capacity of medium-to-large molecules may be effective in cytokine storms involving inflammatory mediators, such as IL-6 [9,10]. ATA membranes may be useful in severe rhabdomyolysis, during which myoglobin, a substance with a molecular weight similar to that of IL-6 (approximately 17,000 Da), is the key component, and blood purification therapy is frequently performed to remove myoglobin [11]. Albumin is an essential protein, and its frequent loss is a disadvantage. The estimated 24-hour albumin loss was approximately 22 g, assuming a plasma albumin concentration of 2.0 g/dL. Although this value is relatively small compared with the results of previous studies using polysulfone membranes [12], the loss of albumin represents an important protein loss. Albumin loss associated with ATA membrane use remains an important issue that requires real-world validation in future studies.

This study had some limitations that need to be addressed. First, although human blood was used, this was an ex vivo study and did not consider actual clinical symptoms. Atan et al. performed continuous hemodiafiltration using a high-cut-off membrane and reported that although cytokines were removed efficiently, the cytokine concentration in blood did not decrease [13]. However, the ATA membrane used in this study had a higher cytokine removal efficiency than existing hemodiafiltration devices, and its effectiveness needs to be confirmed under actual clinical conditions. Second, the filtration flow rate was limited to 2000 mL/hour. In this study, only flow rates that were deemed to approximate actual clinical conditions were investigated. The ATA membrane-based hemofilter used in this study was more efficient than existing ones; therefore, it is necessary to confirm the optimal filtration flow rate under actual clinical conditions. Third, morphological evaluations were not performed. The ATA membrane has an asymmetric dual-layer structure consisting of a thin dense layer and a rough support layer. It is assumed that a thinner, denser layer achieves a more homogeneous pore size, resulting in more sensitive fractionation characteristics; however, this has not been proven morphologically. Morphological evaluation using electron microscopy will be required in the future. Fourth, all blood samples in this study were obtained from male donors. Although the effect of sex on cytokine induction in this system is unknown and may have influenced cytokine concentrations, this study primarily evaluated the removal efficiency of specific cytokines and albumin. Thus, the impact of the male-only samples on the results was considered minimal. Finally, this study was conducted using an ex vivo design and measured only one cytokine (IL-6); therefore, the effects of ATA membranes on other inflammatory cytokines in vivo remain unclear.

## Conclusions

The results of this study suggest that compared with conventional CTA with a homogeneous membrane structure, ATA with the target structure may possess a markedly higher IL-6 removal capacity while also exhibiting greater albumin loss. The ATA membrane was associated with a substantially lower change in the intracircuit pressure over 24 hours than the CTA membrane. Future studies are required to validate the removal capacity and durability of ATA membranes under real-world clinical conditions.
